# Factors Influencing Turnover Intention among Male Nurses in Korea

**DOI:** 10.3390/ijerph18189862

**Published:** 2021-09-18

**Authors:** Su Ol Kim, Sun-Hee Moon

**Affiliations:** 1 Department of Nursing, Kwangju Women’s University, Gwangju 62396, Korea; teddy627@hanmail.net; 2 College of Nursing, Chonnam National University, Gwangju 61469, Korea

**Keywords:** male nurses, personnel turnover, job satisfaction, organization, regression analysis

## Abstract

The study examined predictors of male nurse turnover intention in Korea using data collected from Korean hospitals. The results were obtained based on a secondary analysis of data previously collected from 306 male nurses in 16 regions of Korea from December 2014 to February 2015. Our findings suggest that male nurse turnover intention is predicted by (1) individual factors: single (B = 0.93, *p* = 0.008); (2) organizational factors: organizational commitment (B = −0.36, *p* < 0.001), job satisfaction (B = −0.27, *p* = 0.001), and job stress (B = 0.24, *p* < 0.001); and (3) social factors: hospital location in medium-categorized cities (B = 0.70, *p* = 0.012) and kinship responsibility (B = 0.13, *p* = 0.026). These factors accounted for 56.9% of the total variance. To lower the rate of turnover intention among male nurses, strategic interventions should be implemented based on the factors identified in this study.

## 1. Introduction

Men’s role in nursing has steadily expanded due to new employment opportunities in the medical field and generally high employment rates [1,2]. For example, the proportion of male registered nurses has increased in both the United States and Australia, from 6.6% in 2013 to 9.1% in 2017 and 8.8% in 2011 to 10.4% in 2014, respectively [2,3]. In Korea, the nursing profession was first opened to males in 1962 and has steadily grown for this demographic, notably growing from 3.8% in 2015 to 5.7% in 2020 [4]. The increasing trend of male entry into nursing can be attributed to employment stability and wages. The average annual salary of male workers in Korea in 2012 was 37 million won (approximately 32,700 USD), while that of nurses in 2015 was 36.3 million won (approximately 32,000 USD), showing a similarity in the salary levels [5]. In addition, the lack of nursing staff in Korea has lowered the risk of dismissal, providing an opportunity for more male workers to pursue a nursing career [6]. Since the increase in diversity caused by the influx of male nursing manpower has positive effects, such as increased leadership [7], efforts for retention of male nurses should be continued.

Nurse turnover has been regarded as a major problem in the nursing field. The high turnover rate of nurses leads to a decrease in the quality and quantity of patient care, which affects patients’ health. Due to the high nurse turnover, patients cannot be served consistent and high-quality care, which leads to increased hospital infections, hospitalization, and mortality [8,9,10]. Because of the turnover of proficient nurses, the remaining nurses develop physical problems due to increased work intensity and extended working hours, as well as psychological problems including reduced job satisfaction and demoralization [10,11]. Therefore, to reduce the negative effects of nurse turnover, a multilateral study on turnover intention is needed. Previous studies suggest that the pattern of turnover of nurses varies according to gender; specifically, male nurses’ turnover intention was shown to be higher than that of female nurses [12,13]. In a study of male nurses in Taiwan, the turnover rates within 1 and 5 years were 23.6% and 44.7%, respectively [14]. Therefore, it is necessary to find gender-specific approaches and solutions to the nurse turnover problem.

In the field of nursing, which is socially recognized as a female occupation, several qualitative studies have demonstrated factors affecting male nurses’ turnover intentions, specifically, stereotype and job stability [6,7,15,16]. On the other hand, valid quantitative studies considering various variables were insufficient in their attempts. Some quantitative studies have reported that organizational commitment, job satisfaction, job stress, burnout, and role conflict affect male nurse turnover intention [1,14,17]. However, these quantitative studies were limited by small sample sizes and a lack of focus on social factors, which are critical areas of concern for male nurses. This study was designed to add to the literature by comprehensively identifying and predicting the factors affecting turnover intention among male nurses in Korea based on a nationwide sample. As such, this study aimed to predict turnover intention among male nurses in Korea by examining not just the influence of individual and organizational factors, but also of social factors.

## 2. Literature Review and Hypotheses

### 2.1. General Categories Influencing Job Turnover

General categories pertaining to nurses’ turnover intention can be divided into individual and organizational factors [18], with those at the individual level including sex, age, education, and marital status, and those at the organizational level including managerial style, empowerment, role recognition, workload, job stress, job dissatisfaction, and supervisory support [1,18,19,20]. These two classes of factors have been shown to influence turnover intention either positively or negatively. Individual factors including younger age, low salary, short career, being single, and lower education and organizational factors including workload, job stress, and dissatisfaction were reported to increase turnover intention [18,20]. On the other hand, organizational factors including organizational commitment, empowerment, job satisfaction, and supervisory support are reported to lower turnover intention [18,20]. While these individual- and organizational-level factors affect the turnover intention of both male and female nurses, their strength appears to be stronger for male nurses. Some studies reported the turnover rate of male nurses to be higher than that of women, and in Taiwan and the USA specifically, the turnover rate of male nurses was reported to be double that of female nurses [14,21,22]. In a study on Italian nurses, it was reported that male nurses’ turnover intention was 1.232 times higher than that of female nurses [23]. Therefore, it is necessary to additionally verify whether other unique factors, other than individual- and organizational-level factors, influence the turnover of male nurses.

### 2.2. Unique Category Affecting Turnover Intention for Male Nurses

The turnover intention of male nurses is particularly influenced by unique social-level factors. These social factors include the gender role conflict experienced by male nurses, the existing stereotype against males working as nurses, and men’s responsibility to support their families [17,24]. In fact, in a study on male nurse turnover experiences, the main factor was found to be the desire to find a stable job, which is related to their need to support their families [6].

The gender stereotype experienced by male nurses differed according to cultural background. In Western culture, gender stereotypes for male nurses tended to be strong [16,25]. Studies in Canada, the USA, and Norway have reported that male nurses are considered gay or to have strong feminine tendencies [16,25,26]. In Eastern cultures, traditionally dominated by Confucian thought, including Korea, there is strong pressure from the family and surroundings that nursing is not a male occupation [27,28]. In a qualitative study of male nurses in Korea, male nurses were rejected when attempting to conduct health care procedures including enema, foley catheter insertion, pressure sore assessment, and so on [29]. Male nurses were unable to adapt to the limitations of patient care and to the working environment centered on female nurses, resulting in a decrease in job satisfaction [6,29]. This decline in job satisfaction became a driving reason for resignation. In recent studies on male nurses’ turnover experiences in Korea and China, job stability and responsibility for their families are emerging as factors affecting turnover [6,28]. It is suggested that the desire to find a stable job in a society where competition is fierce has led to the turnover of male nurses.

## 3. Materials and Methods

### 3.1. Design

This descriptive study investigated the nature of turnover intention among male nurses in Korea, with a particular focus on the factors of job satisfaction, organizational commitment, kinship responsibility, role conflict, and several other characteristics.

### 3.2. Instruments

Based on previous studies, we compiled a list of general characteristics related to individual, organizational, and social factors. More specifically, this included 12 items: age, marital status, education level, total nursing experience, nursing experience in the current workplace, role model as a male nurse, hospital size, position, shift pattern, space for male nurses, annual salary, and hospital location. Most items were variously measured based on short-answer or multiple-choice questions, while the degree of job stress was measured based on a 10-point Likert scale.

Job satisfaction refers to one’s attitude toward one’s job. In this study, it was measured based on a modified version of a previous index [30,31], which specifically consisted of four questions without sub-factors. The responses were measured on a 5-point scale ranging from 1 (strongly disagree) to 5 (strongly agree); total possible scores could therefore range from 4 to 20, with higher scores indicating higher job satisfaction. At the time of development, the scale received a Cronbach’s α of 0.87 [30], and later, it received 0.76 in a study by Kim et al. conducted in 1996 [31]. In this study, it received a Cronbach’s α of 0.84.

Organizational commitment refers to the will or attitude to remain with a given organization due to a strong belief in its practices [32]. In this study, organizational commitment was measured using a scale developed by Mowday and Steers in 1979 and modified by Kim et al. in 1996 [31,32]. The scale consisted of seven questions without sub-factors, with responses measured on a 5-point scale ranging from 1 (strongly disagree) to 5 (strongly agree); total possible scores could therefore range from 7 to 35, with higher scores indicating higher organizational commitment. At the time of development, the scale received a Cronbach’s α of 0.90 [32], and later, it received a 0.82 in a study by Kim et al. [31]. In this study, it received a Cronbach’s α of 0.87.

Kinship responsibility is one’s degree of economic and mental obligation to one’s family, whom one has to support [33]. In this study, kinship responsibility was measured using a tool developed by Price and Muller [33]. The scale consisted of four questions without sub-factors. The responses were measured on a 5-point scale ranging from 1 (strongly disagree) to 5 (strongly agree); total possible scores could therefore range from 4 to 20, with higher scores indicating higher kinship responsibility. In Price and Muller’s study, the Cronbach’s α was 0.83 [33], and in this study, the Cronbach’s α was 0.72.

Role conflict refers to conflict that occurs when one’s job role differs from one’s beliefs [34]. Here, role conflict was determined using a tool developed by Rizzo et al., which consisted of four questions based on a 5-point Likert scale wherein higher scores indicate higher role conflict [34]. In the study by Rizzo et al., the Cronbach’s α was 0.82 [34], and in this study, the Cronbach’s α was 0.81.

Turnover intention is a strong predictor of actual turnover [35]. In this study, it was measured using a scale developed by Kim et al. [31], which specifically consisted of four questions without sub-factors. The items were rated using a 5-point scale ranging from 1 (strongly disagree) to 5 (strongly agree); total possible scores could therefore range from 4 to 20, with higher scores indicating higher turnover intention. At the time of development, the scale received a Cronbach’s α of 0.85 [31]. In this study, it received a Cronbach’s α of 0.88.

### 3.3. Sample and Data Collection

In this study, we analyzed data previously obtained from all regions in Korea using a model for turnover intention among male nurses, specifically including one megacity (Seoul), six major cities (Busan, Incheon, Daegu, Gwangju, Daejeon, and Ulsan), and nine states (Gyeonggi, Gangwon, Chungbuk, Chungnam, Gyeongbuk, Gyeongnam, Jeonbuk, Jeonnam, and Jeju) [36]. The inclusion criteria were as follows: (1) male nurses who had worked at their current hospitals for more than 3 months and (2) gave their consent to participate [36].

The data collection process was conducted at 60 medical institutions nationwide from December 2014 to February 2015. We visited hospitals that allowed participation in the study and identified a list of male nurses who met the inclusion criteria. The nursing staff informed the male nurses about the purpose of the study and the participation process, and then identified the nurses’ willingness to participate in the questionnaire. We distributed paper-based self-report questionnaires to the male nurses who consented to participate in the study. Specifically, the questionnaires were distributed to 330 male nurses, and we received 312 responses (a response rate of 94.5%). Of those, six were excluded due to missing answers. Therefore, the study was conducted on 306 (92.7%) of the responses.

### 3.4. Ethical Approval

This study received approval from the Changwon National University Institutional Review Board (deliberation waiver No. 7001066-202007-HR-014). The original data were also approved by the institutional review board of Ewha Womans University (No. 82-4). All participants willingly engaged in the original data collection process and signed written informed consent forms.

### 3.5. Data Analysis

All data were analyzed using the IBM SPSS software, version 26.0. All variables were analyzed based on frequencies, percentages, or means. Following this, differences in turnover intention based on general characteristics were analyzed via the *t*-test or one-way analysis of variance. Finally, a multiple linear regression was conducted to determine the factors that affected turnover intention among the respondents. The input independent variables that were selected included nine continuous variables (organizational commitment, job satisfaction, annual salary, age, total nursing experience, nursing experience in the current workplace, kinship responsibility, job stress, role conflict) and eight categorical variables (marital status, education, hospital location, hospital size, position, shift pattern, space for male nurses, role model as a male nurse). The categorical variables were converted into dummy variables for analysis. Backward elimination was used to select variables used in the multiple linear regression. Backward elimination is effective when there are many variables, and it is the most conservative method of variable selection [37]. All independent variables were entered into the regression analysis first, and each one was deleted, one at a time, if the variable did not contribute to the regression equation. Finally, a model consisting of six variables was selected through backward elimination.

## 4. Results

The average participant *age* was 28.31 ± 3.68, with 220 (71.9%) participants aged 20 to 30 years, and 248 (81.0%) reporting their marital status as single (Table 1). Of the 306 participants, 100 (32.7%) reported having a total of between 1 and 3 years of nursing experience, while 114 (37.3%) said they had been working in their current departments for between 1 and 3 years. Two hundred and eighteen (71.2%) participants said there were no role models for male nurses. Next, 210 (68.6%) of them said that they were mainly in charge of general nursing work, while 172 (56.2%) worked in shifts. Two hundred and thirty-three (76.1%) participants worked in hospitals with spaces for male nurses only. Regarding compensation, 102 (33.4%) had an annual salary of less than 30 million won (approximately 27,000 USD), with only 12 (3.9%) earning more than 50 million won (approximately 46,000 USD). The hospitals in which participants worked were mainly located in medium-categorized cities with populations ranging from 1 to 5 million.

The individual factor variables that showed significant differences with turnover intention were marital status (t = 6.08, *p* = 0.014) and role models as a male nurse (t = 19.25, *p* < 0.001). The turnover intention of participants in their 20s was higher than that of those in their 30s (F = 3.32, *p* < 0.037). Participants with diplomas showed higher turnover intention than those with master’s degrees (F = 5.53, *p* = 0.004), and those with 1 to 5 years of total nursing experience showed higher turnover intention than all others (F = 7.63, *p* < 0.001). Regarding experience in the current workplace, participants with 1 to 3 years of experience showed higher turnover intention than those with over 5 years (F = 4.52, *p* = 0.004). As for annual salary in the organizational factors, those who earned over 50 million won (approximately 46,000 USD) showed lower turnover intention than those who earned less than 50 million won (F = 3.48, *p* = 0.004). Hospital location in medium-categorized cities showed higher turnover intention than those in small and large cities (F = 9.33, *p* < 0.001).

The means of the variables were as follows (Table 2). The average of turnover intention was 12.50 ± 3.43, while it was 22.93 ± 4.52 for organizational commitment, 12.98 ± 2.80 for job satisfaction, 6.17 ± 2.14 for job stress, 11.89 ± 2.29 for kinship responsibility, and 10.87 ± 2.72 for role conflict. The skewness and kurtosis of all the variables were both within ±2. The relationship between turnover intention and organizational and social factors was assessed. Based on the result, turnover intention was significantly negatively related to organizational commitment (Pearson’s r = −0.69) and job satisfaction (Pearson’s r = −0.66). On the other hand, turnover intention was significantly positively related to job stress (Pearson’s r = 0.39) and role conflict (Pearson’s r = 0.31).

A multiple linear regression model was used to predict turnover intention based on comprehensive variables (Table 3, Equation (1)). To perform regression analysis, autocorrelation of the dependent variable and multicollinearity between independent variables were assessed. The Durbin–Watson value of the selected regression model was 1.88; there was no autocorrelation. The tolerance value was 0.36–0.95, and the variance inflation factor (VIF) was 1.05–2.80, so there was no multicollinearity. The independent variables were found to be statistically significant with single (B = 0.93, *p* = 0.008), organizational commitment (B = −0.36, *p* < 0.001), job satisfaction (B = −0.27, *p* = 0.001), job stress (B = 0.24, *p* < 0.001), hospital location in medium-categorized cities (B = 0.70, *p* = 0.012), and kinship responsibility (B = 0.13, *p* = 0.026). A significant regression equation was found (F = 65.85, *p* < 0.001), with an R^2^ of 0.569. Participants’ predicted turnover intension was equal to 20.17 + 0.93 (single) − 0.36 (organizational commitment) − 0.27 (job satisfaction) + 0.24 (job stress) + 0.70 (hospital location in medium-categorized cities) + 0.13 (kinship responsibility).

F (*p*) = 65.85 (*p* < 0.001), R^2^ = 0.569, Adjusted R^2^ = 0.561.

Equation (1). Regression equation for predicting turnover intention.

Turnover intention = 20.17 + 0.93 (Single) − 0.36 (OC) − 0.27 (JS) + 0.24 (Job stress) + 0.70 (HMC) + 0.13(KR)(1)

Abbreviations: OC, organizational commitment; JS, job satisfaction; HMC, hospital location in medium-categorized cities; KR, kinship responsibility.

## 5. Discussion

The proportion of male nurses is increasing across the globe [2,3]. However, it is still relatively low in Korea, especially considering the instability of the overall workforce numbers [4,6], thus warranting continued research into turnover intention. Since turnover is different according to gender, a strategic approach is necessary to lower male nurses’ turnover intention. As such, this study added to the literature by comprehensively identifying and predicting the factors affecting turnover intention among male nurses in Korea. We hope that this study will be utilized to design specific strategies to lower male nurse’s turnover intention.

Our study has three theoretical implications and five practical implications, and the specifics are as follows.

### 5.1. Theoretical Implications

First, in order to predict a stable balance between supply and demand in the nursing workforce, it is first necessary to identify turnover intention and then analyze the factors that affect it. In a previous study on turnover intention among nurses, 70% of respondents who answered that they intended to leave their jobs actually did [35]. This shows that turnover intention is a strong predictor of the real turnover rate. In this context, preventive interventions should focus on identifying the factors that influence turnover intention [35,38,39].

Second, concerning education level, this study also found that participants with diplomas showed higher turnover intention than those with master’s degrees. This is consistent with previous research involving male nurses in Korea, which found lower education levels to be associated with higher turnover intention [1]. Further, Zhang conducted interviews with male nurses to investigate their working experiences, finding that the reasons for remaining with organizations included promotion opportunities, stable employment, and salary, while reasons for leaving included lack of interest, heavy workloads, and family and societal pressures. Another study on the turnover experiences of Korean male nurses found that “seeking a stable place” constituted a major category [6]. Based on these findings, male nurses may find increased job stability by obtaining advanced degrees, working to increase self-esteem, and seeking opportunities for promotion.

Third, in this study, organizational commitment and job satisfaction were found to have significant negative effects on turnover intention. These results were consistent with several reports showing that both factors had strong negative effects on turnover intention [18,20]. Further, this study found that organizational commitment had a stronger negative effect than job satisfaction. The difference in levels of influence presented by these variables can also be seen when looking at meta-studies on Korean journals. For example, Lee and Kang found that the effect size of organizational commitment (Esr = −0.63) was larger than that of job satisfaction (Esr = −0.49) [40]. While no meta-analyses are available on turnover intention among this demographic in other countries, a review study found that job satisfaction was reported more often than organizational commitment [18]. As such, unique sociocultural environments may be responsible for the different levels of influence found for organizational commitment and job satisfaction on turnover intention. Further, since turnover intention, job satisfaction, and organizational commitment are related to both organizational culture and the social environment [1,18,28], the link between turnover intention and behavior is influenced by the cultural factor of individualism [41]. Because Korean culture emphasizes communal behaviors, it can be interpreted that organizational commitment (which represents organizational attachment) has a greater influence on turnover intention throughout the country.

### 5.2. Practical Implications

First, this study further analyzed the differences in turnover intention based on salary and found that the high-salary group showed decreased turnover intention. However, in the regression analysis results, annual salary has been removed. While previous studies have shown that low salaries are generally important factors in the context of actual turnover [22,42], a recent review also reported that annual salary was less influential than job satisfaction, organizational commitment, job stress, and burnout [18,40]. Notably, annual salary only has a small influence on turnover intention among male nurses in Asian cultures, where economic responsibility is generally considered important (i.e., maintaining consistent “breadwinner” status). In this study, most participants were single, which may have also affected the results. On the other hand, most may have simply placed more importance on factors such as organizational growth, job satisfaction, and organizational commitment, particularly when compared to the material rewards of employment.

Second, in this study, hospital location in medium-categorized cities was also an important variable. While no previous studies have directly investigated differences in turnover intention based on city size, some have shown higher turnover intention among nurses working in general hospitals when compared to those working in hospitals located in small cities [20]. This study found that participants working in medium-sized cities (population of 1 to 5 million) had higher turnover intentions than those in small and large cities. Relatively small cities tend to be conservative; hence, the stereotype toward male nurses may be stronger. This conservative social atmosphere may have persuaded the male nurses to move to more liberal cities, thus increasing their turnover intention. In this regard, participants living in medium-sized cities may have sought opportunities to move to small or large cities. However, there are many limitations to this interpretation, meaning that more detailed research is needed.

Third, in this study, kinship responsibility was predicted as a factor that increased male nurses’ turnover intention. In a turnover study of nurses (including men and women) in the USA, it was reported that the turnover rate of married nurses with children was 2.85 times higher than that of single nurses without families [43]. Since there is no study that examines the relationship between kinship responsibility and turnover intention targeting only male nurses, a detailed comparison of this result with those of other studies is not possible. However, a social atmosphere in which men are generally regarded as the heads of their households may increase the kinship responsibility of male nurses, which may become a reason to seek a more stable job and higher-salary workplace.

Fourth, role conflict and job stress increase nurse turnover intention (for both men and women) [18]. Although the difference between job stress and role conflict according to gender could not be confirmed in previous studies, it has been reported that role ambiguity and conflict lead to job stress in male nurses, and when this situation persists, they often consider resigning [6,22]. Therefore, in order to reduce male nurses’ turnover, it is necessary to establish a strategy that considers the causes of job stress and gender role conflict.

Fifth, when examining the differences based on individual factors, turnover intention was found to be higher among those reporting their marital status as single. This contradicts previous research reporting no differences in turnover intention among male nurses in Korea based on marital status [1]. On the other hand, a review study on turnover intention among nurses in general (including men and women) found a high rate of turnover intention among the single group [18], which supports the current results. It is also important to note that Ahn et al. surveyed a relatively small sample in limited areas of Korea. Since this study recruited more than 300 participants from all regions of Korea, it can be said that the characteristics of male nurses were better reflected. In this study, the single male nurse factor was an important predictor of turnover intention. In a qualitative study on male nurses’ turnover experience, “seeking a stable place” was the core factor [6]. Male nurses with a single status in this study showed an intention to change jobs in order to seek a stable workplace, while those with a married status showed a low turnover intention because they have already settled in a stable hospital.

### 5.3. Limitation

This study had a limitation in that the data collection for this study took place in 2015. Nonetheless, because this study targeted male nurses, who are increasing in proportion in the nursing workforce, and more than 300 individuals were recruited on a national scale, this study still presents a unique contribution to the current literature. Notably, 56.9% of the variance in turnover intention among Korean male nurses can be explained by the variables selected in this study. Future interventional studies aimed at lowering turnover intention among this demographic can therefore be planned around those variables.

## 6. Conclusions

This study confirmed that individual, organizational, and social factors affected turnover intention among male nurses in Korea. In this regard, 56.9% of the total variance was explained by being single, organizational commitment, job satisfaction, job stress, hospital location in medium-categorized cities, and kinship responsibility. Management strategies aimed at predicting and lowering turnover intention among these individuals should therefore consider these and other influential factors.

## Figures and Tables

**Table 1:** General characteristics and differences in turnover intention (*N* = 306).

Factors		Categories	*n* (%)	Turnover Intention			
				Mean	Standard Deviation	t or F	*p* (Scheffe)
Individual	Age (years)	20~29 (a)	220 (71.9)	12.82	3.36	3.32	0.037 * (a > b)
		30~39 (b)	83 (27.1)	11.72	3.50		
		≥40 (c)	3 (1.0)	11.33	4.04		
	Marital status	Single	248 (81.0)	12.74	3.36	6.08	0.014 *
		Married	58 (19.0)	11.51	3.58		
	Education	Diploma (a)	170 (55.6)	13.00	3.40	5.53	0.004 * (a > c)
		Bachelor’s (b)	128 (41.8)	12.02	3.37		
		≥Master’s (c)	8 (2.6)	9.87	2.99		
	Total nursing experience (years)	<1 year (a)	79 (25.8)	11.78	3.14	7.63	<0.001 *** (a,d < b,c)
		1 to less than 3 (b)	100 (32.7)	13.26	3.67		
		3 to less than 5 (c)	63 (20.6)	13.48	3.27		
		≥5 (d)	64 (20.9)	11.28	3.03		
	Nursing experience in the current workplace (years)	<1 year (a)	94 (30.7)	11.87	3.30	4.52	0.004 ** (d < b)
		1 to less than 3 (b)	114 (37.3)	13.26	3.54		
		3 to less than 5 (c)	53 (17.3)	12.87	3.20		
		≥5 (d)	45 (14.7)	11.51	3.30		
	Role model as a male nurse	Presence	88 (28.8)	11.19	3.44	19.25	<0.001^***^
		Absence	218 (71.2)	13.04	3.29		
Organizational	Hospital size (beds)	Small (<100)	19 (6.2)	13.68	4.48	1.85	0.158
		Medium (100 to <500)	104 (34.0)	12.74	3.19		
		Large (≥500)	183 (59.8)	12.25	3.42		
	Position	Nurse	210 (68.6)	12.48	3.53	0.02	0.977
		Physical assistant	91 (29.7)	12.57	3.22		
		Nurse manager	5 (1.6)	12.60	3.04		
	Shift pattern	Day shift only	134 (43.8)	12.38	3.34	0.33	0.562
		Shift work	172 (56.2)	12.61	3.50		
	Space for male nurses	Presence	233 (76.1)	12.35	3.37	0.40	0.151
		Absence	73 (23.9)	13.01	3.62		
	Annual salary (million won units)	<30 (a)	102 (33.4)	13.04	3.70	3.48	0.004 ** (a,b,d > f)
		30 to less than 35 (b)	86 (28.1)	12.86	3.31		
		35 to less than 40 (c)	60 (19.6)	11.78	3.13		
		40 to less than 45 (d)	31 (10.1)	12.84	3.07		
		45 to less than 50 (e)	15 (4.9)	11.53	3.16		
		≥50 (f)	12 (3.9)	9.50	2.78		
Social	Hospital location (millions of people)	Small city (<1 million) (a)	109 (35.6)	12.12	3.48	9.33	<0.001 *** (a,c < b)
		Medium city (1 to <5 million) (b)	115 (37.6)	13.54	3.14		
		Large city (≥5) (c)	82 (26.8)	11.58	3.43		

Abbreviations: * *p* < 0.05, ** *p* < 0.01, *** *p* < 0.001.

**Table 2:** Descriptive statistics and correlations (*N* = 306).

Variables		Mean	Standard Deviation	Turnover Intention	Organizational Commitment	Job Satisfaction	Job Stress	Kinship Responsibility
Turnover intention		12.50	3.43	1				
Organizational	Organizational commitment	22.93	4.52	−0.69 **	1			
	Job satisfaction	12.98	2.80	−0.66 **	0.77 **	1		
	Job stress	6.17	2.14	0.39 **	−0.29 **	−0.42 **	1	
Social	Kinship Responsibility	11.89	2.29	−0.03	0.15 **	0.08	−0.02	1
	Role conflict	10.87	2.72	0.31 **	−0.31 **	−0.30 **	0.21 **	0.09

Abbreviations: * *p* < 0.05, ** *p* < 0.01.

**Table 3:** Multiple linear regression results (*N* = 306).

Variables		B	SE	Beta (β)	t	*p*
Constant		20.17	1.20	-	16.78	<0.001
Individual	Single	0.93	0.35	0.11	2.68	0.008
Organizational	Organizational commitment	−0.36	0.05	−0.48	−7.93	<0.001
	Job satisfaction	−0.27	0.08	−0.22	−3.41	0.001
	Job stress	0.24	0.07	0.15	3.52	<0.001
Social	Hospital location in medium-categorized cities (1 to <5 million)	0.70	0.28	0.10	2.54	0.012
	Kinship responsibility	0.13	0.06	0.09	2.24	0.026

## Data Availability

The data that support the findings of this study are available from the corresponding author upon reasonable request.
